# Artificial Dentistry

**Published:** 1873-12

**Authors:** H. L. Sage


### DENTAL REGISTER.

Vol. XXVII.] DECEMBER, 1873. [No. 12.

### ARTIFICIAL DENTISTRY.

BY H. L. SAGE, D. D. S.

*Read before the Connecticut State Dental Association, October 14th, 1873.*

The practice of artificial, or mechanical dentistry, as it is termed, is not now-a-days so intimately blended with that of operative, business-wise, as in the past.

That is, the expert operator will usually be found to confine his attention and best efforts more particularly to the preservation of the natural teeth, and the treatment of diseased conditions of the oral cavity, with a certain degree of pride in this direction which he does not entertain in regard to the practice of the merely artificial branch, owing in part to the fact that the latter has, in a great measure, fallen into the hands of the so-called "cheap dentist;" a certain tendency or proclivity of human nature so prevalent with the masses, to obtain the lowest rates, being taken advantage of by many practitioners, to the exclusion of all that is consistent with the true elevation of this branch of dentistry ; so that the most conscientious and skillful in the profession are obliged to secure a higher rate of compensation than is demanded by a less talented, or, at least, a less aspiring and pains-taking class, for products of less practical and intrinsic value than are obtained by the use of more expensive materials, and the employment of more time, taste and ability : so that a limited practice, so far as mechanical dentistry is concerned, will usually be retained by the specially careful and expert operator, *and* mechanical dentist, and for the reason, also, that many, whose necessities call for dental substitutes, not being able to discriminate between the highest creations of artistic taste and the crude productions, or unwilling to bear the greater expense of procuring the former, are satisfied with the inferior article ; and it does not help the matter to affirm that the fault lies with the profession.

While it is true, that the cheap specialist will usually be found to be quite deficient in the essentials that constitute a finished operator upon the natural organs, many of the latter class are possessed of qualifications which enable them to practice both branches, with credit to themselves, and honor to the profession.

Modern artificial dentistry consists, with the majority of dentists, in the construction of plates from red rubber principally, and from metal secondarily—or with many, from the former exclusively—and the mounting of porcelain teeth thereon, in the shortest, cheapest and most expeditious manner possible, without much regard to the display, in any essential degree, of artistic taste.

But the Ceramic Art, as developed in the manufacture of porcelain teeth, has come to be so nearly separate and distinct from that of mechanical dentistry proper as to rank as an entirely different, and, as now carried on, *higher* branch of the business, in which more capital, skill and taste, have been, and are being displayed ; so that while one branch appears to retrograding, the other is steadily advancing in excellence, and towards perfection, though there are yet higher planes to be reached.

That they are separate is well, perhaps better than if it were otherwise; but it is evident that the two should keep pace with each other, in order to develop the "perfect whole," or, in other words, the productions of the skilled manufacturer of teeth, be they ever so faultless in everything that pertains to natural appearance, durability and practical value, must be appropriately selected, adapted and mounted, with the idea of general fitness fully carried out,—with an almost imperceptible blending of nature and art, would we secure the full benefits of the products or results of the two branches of artificial dentistry. For, be the porcelain moulded into faultless forms, shaded with tints true to nature, and adapted to all the requirements of taste, the desired effect may be lost, or productive even of an *appalling hideousness* of expression, by a lack of care or ability on the part of the dentist, or by the mere *mechanical* manifestations of a crude, uncultured taste.

Time was, when his duties were more complicated and varied than at present; when a higher order of talent was required than is now *absolutely* necessary, in order to conduct a practice; when it was essential to be at once a manufacturer of teeth and a good plate-worker—plate-worker being a term applied to one who was competent to smelt, refine and convert into plates, the precious metals; strike up, fit and adapt them to the mouths of patients; grind, arrange and attach thereto the teeth; and finish in a manner that was charming to behold.

Then, too, block carving was not a rare accomplishment. When blocks were not carved to suit a particular case, single teeth were employed, and the mechanically stiff and artificial appearance was not nearly so marked as it has been since the introduction of rubber, and, with its employment, the precisely-arranged and rigid-looking moulded blocks, with the preferences which obtain with many rubber workers and their patients, for snow-white teeth, carried out to the letter; those which can be the most easily arranged being selected, while *shade*, style and general adaptation or fitness are ignored.

While Art and Mechanism were in the olden time combined, Mechanism alone is now too often the sole presiding Genius of the laboratory ; *i. e.* where the *base* rubber base is alone or chiefly employed, and then Mechanism is too apt to be of a very low grade as well, while the pure, healthful, life-like and beautiful continuous gum work scarcely, or only in exceptionable cases, finds a place in the list, but with which no other style of work is at all comparable, or has been found to hold so high a place in the niche of dental art and mechanism.

"When'er we take our walks abroad," our observing eyes will detect numerous specimens of inferior and unnatural looking artificial dentures ; or go into any company of matrons where "false sets" do most abound, and even the eye of a novice may distinguish the artificial character of that portion of the *smile* which is dependent upon porcelain for a *finish*, and which is so apt to lack even an approximation to that beauty of expression which Nature's touch imparts.

Exceptional cases there are, let us state for the credit of our profession—but that the art occupies a lower place than formerly, cannot be denied ; and why, can be answered by inference, if the foregoing statements are in anywise sustained by facts.

We may infer, as before intimated, that one of the causes of this is the cheaper and *baser* character of the materials employed as bases for artificial teeth, than which none has been so largely used, or is in itself so vile a compound *when* used for this purpose, as the material *called* "red rubber." If it does not *morally* debase the dentist that inserts teeth upon it, it *physically* debases the one who wears it, oftentimes at least, the proof of which does not seem to be lacking.

Moreover, mounting a set of teeth upon rubber is so easily accomplished, after a sort, any how, or in tolerable style, that the introduction of the compound has been the occasion for the advent of a large number of ready-made, "first-class" dentists, whose wonderful genius has excited the admiration of a large and constantly increasing number of toothless and would-be toothless indviduals ; for he who can insert artificial teeth that are better than natural ones ever were or can be, that have advantages that natural ones never did or can possess, and at such low prices too, is certainly a genius, and a man who "goes about doing good," in the highest sense of the term. But so long as the poison incorporated in the material is slow and insidious, and its work not always apparent to the senses, and people rest in fancied security, does it matter *much?* For is it not better to die and not know it, than to live and be aware of it? "Where ignorance is bliss 'tis folly to be wise."

As a final answer to the question, what has degraded the practice of artificial dentistry, this seems an appropriate reply: the introduction of cheap and deleterious materials, requiring little labor and skill in their employment—inducing the advent of many inferior men into the profession—lowering the standard of professional excellence—debasing the public mind and driving out much of the best talent into the more congenial field of operative dentistry, to the entire exclusion of the mechanical branch from their practice. Aside from this, the natural organs respond to abuse and bad practice, while the effects of good treatment also, they are not slow to indicate; and thus the difference between the two modes of operating is apparent to the *senses*, if not to the intelligence of the public, —while it is not easy, or quite so important. to distinguish between the crude and the finer productions of mechanical dentistry, for the reason that the former constitute by far the larger portion of those subjected to public scrutiny; and hence a certain style becomes familiar, and when that style preponderates the eye becomes educated or accustomed thereto, and finally comes to tolerate it, so that what was once a painful apparition, becomes a "thing of beauty." "We first endure, then pity, then embrace."

The question, what can be done to elevate the practice of artificial dentistry, has been partially and inferentially answered in the foregoing remarks. Without impugning or calling in question the motives of a worthy and conscientious class, who may honestly differ in regard to the employment of a certain base for artificial teeth, or its local or constitutional effects upon the wearer, these suggestions in relation thereto, have been offered.

There are few, if any, harmless substances in use as bases, that can be afforded as cheaply as rubber, which, notwithstanding its advantages, is not, in all cases at least, innocuous and healthful. If the last objection should not banish it entirely from the list of bases, it should lead to greater discrimination in its employment; and as it is not in all cases easy to determine, without a trial, who may be injured by wearing it—or even then, so insidious may be its effects—it is wise to discard its use entirely. In many cases, at least, it is certainly contra-indicated and there are substitutes without any of the objections that pertain to it, though requiring, doubtless, a higher degree of skill, and more time and labor in their manipulation; and here the tendency would be elevating. Notwithstanding, rubber is an indispensable adjunct in both branches of dentistry, for some special purposes.

But, looking at the question in another aspect, the introduction of cheap bases induces a low asking price; a low asking price presents a temptation to subordinate excellence in dental art, to inferiority in everything that constitutes taste, fitness, durability, etc., and excellence when applied in operative dentistry as well; a low asking price makes *dishonest* practitioners, by withholding one of the motives to honest endeavor, and thus it *must* be, as human nature is constituted, leaving out of view that abstract principle, that benevolence should share in the government of the matter; a low asking price enslaves the body and, where excellence should be sought, the soul; cramps the intellect and dwarfs the faculties; crushes ambition and lessens self-respect, notwithstanding it may aid in the cultivation of a slavish humility; it produces slovenly workers, who practically reverse the old adage, and do nothing "well" which is "worth doing at all;" a low asking price is coupled with the sale of inferior products relatively, whether of nature, or of so-called art; a low asking price is associated with "a competition for cheapness, and not for excellence;" a low asking price is, in fact, a sort of abject slavery.

What are the effects on the patient?

It affords an inducement to neglect the natural teeth. Many reason thus: Artificial teeth are much cheaper than natural ones, which require constant attention. One filling will sometimes cost more than a set of teeth—certainly what is necessary to be done in my mouth will more than pay for a set, a fine set, on rubber ; these never ache, are serviceable, durable, as conducive to health and comfort, and present a more *regular*, a *whiter*, and more attractive appearance, than natural teeth (the latter assertion being true in the case of many, who make the mouth a receptacle for fertilizing materials, instead of daily, habitually and thoroughly removing them, and disinfecting the oral cavity, in accordance with the laws of health, good taste and good breeding). In this respect, he continues, art is better than nature. Dead matter is better than living. Teeth are wanted for mechanical purposes only.

Why were teeth created, he asks ; attached to us like so many pegs ? It was a sad mistake.

But what would infants and children do, if dependent upon metal, rubber and porcelain ? Would not development be retarded, expression lost, voice and speech modified, the tissues of the mouth become diseased, and the performance of many physiological functions checked ? Could a child learn the use of artificial teeth ? How many times would it be necessary to replace them during minority, in order to keep pace with development or so as not to check it, as the Chinese check the growth of their pedal extremities ?

These *inquiries* should be a reflex answer to the question, why were teeth created ?

The question as to whether mechanical dentistry should be practiced as a specialty, is being rapidly solved by the logic of events. In fact, we are drifting towards a separation of the two branches of dental practice, and whatever may be our ideas concerning the matter, in this respect, it is in a fair way to regulate itself.

It would seem that the tendency would be elevating, since the practice of a specialty implies a condensation of the time, talents and energies, and the bending and subjugation of these to a single purpose, the work of a life often being freely given by the enthusiastic and earnest worker, to the unfolding and developing of certain ends, aims and objects, and the pursuit, by observation, study and experiment of all that he is capable of bringing forth to practical use and value in his profession. But uninterrupted progress cannot always be expected : It is only when exigencies may arise to develop it. Fresh discoveries and inventions, for instance, may revolutionize science and art in certain directions, placing it on a higher plane, and thus a more scientific and reasonable practice come to be followed. Or a demand may arise for men better qualified by nature and culture for the positions they may hold, and that demand may come from the profession to which they belong ; for it is not to be expected that the public will remedy what is already within the control of the profession, or even observe failures that the latter may tolerate or look upon with composure, without any concert of action, or attempt to remedy by law or gospel the abuses that may exist.

If artificial dentistry is a meie trade, or a fine art even, it should be so known and designated, and its specific duties defined, and what may be considered requisite acquirements to honorably and successfully prosecute it. What is expected of the candidate for this branch should be determined, and diplomas or certificates furnished well-qualified specialists in the science, *if* it has any claim to *be* a science, or shall be practiced as a specialty. Then shall the two branches of dentistry work unitedly and harmoniousy side by side, each advancing in excellence, because of the higher standard which shall have been set up for attainment, and the consecration and con densation of the forces of mind and matter in these direct ions.

The mission of operative dentistry is pre-eminently to save, and this being the object, it is making rapid progress.

The true mission of artificial dentistry is, to produce a substitute for the lost, and as nearly as possible, *reproduce*, and thus add to life its charms, by indirectly preserving health and ministering to the *comforts* of life.

Without always carrying out this idea, it has, in many instances, sacrificed valuable organs in order to satisfy the caprices of patients, or to supply less valuable substitutes, for a consideration. To sacrifice living, or dead, or partially dead teeth, for purposes of gain simply, or to sacrifice them at all, unless the loss shall bring therewith obviously greater benefits to the loser, which in many cases it does not, is wrong doing and tends to degrade the practice, and has proved a temptation and a snare to many who may not have considered the matter from a moral stand-point. When the principle embodied in the "golden rule" shall govern the motives and actions, the "principles and practice of dental surgery" will find their highest moral, as well as scientific, altitude, and we may reasonably expect that whatever tends to degrade will only come from ignorance, and not from bad motives, while the unceasing evolution of light will so tend to disperse the darkness, that the highest point shall have been reached consistent with our mental and physical infirmities and the lack of that full spiritual development, to which our material bodies seem to be a hindrance.

